# Inclusion of Curcumin in the Diet of Prepartum Cows and Its Effects on Animal Health and Production

**DOI:** 10.3390/vetsci13090944

**Published:** 2026-09-11

**Authors:** Daiane da Silva dos Santos, Aleksandro Schafer da Silva, Angélica Scheid, Gabriela Schroeder, Carine De Freitas Milarch, André Thaler Neto

**Affiliations:** 1 Programa de Pós-Graduação em Ciência Animal, Universidade do Estado de Santa Catarina, Lages 88035-901, SC, Brazil; daiane.zootecnista1993@gmail.com (D.d.S.d.S.); angelica.scheid@edu.udesc.br (A.S.); 2 Programa de Pós-Graduação em Zootecnia, UDESC, Chapecó, Rua Beloni Trombeta Zanini, 680E, Bairro Santo Antônio, Chapecó 89815-630, SC, Brazil; 3 Faculdade IELUSC, Joinville 89201-270, SC, Brazil; 4 Departamento de Produção Animal e Alimentos, Universidade do Estado de Santa Catarina (CAV), Avenida Luiz de Camões, 2090, Bairro Conta Dinheiro, Lages 88520-000, SC, Brazil

**Keywords:** curcumin, transition period, lactation, prepartum, immunity

## Abstract

This study aimed to evaluate whether adding curcumin to prepartum cows’ diets positively affects metabolism, immune response, milk and colostrum production and quality. Forty Holstein cows were divided into two homogeneous groups, with 20 animals per treatment, balanced according to milk production (kg/day) during the previous lactation and parity. The experiment was conducted in a continuous trial, with each groups of animals randomly assigned to one of the treatments: (a) addition of curcumin to the diet (152 mg/day) in the prepartum period; (b) without additive, used as control. The study was carried out on a commercial farm in a compost barn confinement system. Cows were fed with curcumin only during the 21-day prepartum period. Postpartum monitoring, data collection, and sampling were conducted for 28 days without curcumin. After calving, the animals had access to free-flow robotic milking. The variables colostrum production and quality were evaluated, followed by milk analysis. Blood parameters for biochemical, proteinogram and antioxidant analyses were collected at strategic periods of the experiment. We found that the prepartum treatment had no effect on the milk production, composition and physicochemical properties (*p* > 0.05). The serum concentrations of beta-hydroxybutyrate (BHBA), albumin, AST, ALT and GGT of the cows were not affected by the treatments, while the globulin levels were higher in the curcumin group on the seventh day postpartum (*p* = 0.05). The addition of curcumin caused immunomodulation on globulins, where higher serum concentrations of IgA, Ig heavy chain, haptoglobin and transferrin (*p* ≤ 0.001) were observed, as well as lower serum levels of C-reactive protein and ceruloplasmin (*p* ≤ 0.0002). The serum and colostrum oxidant/antioxidant profile did not show any difference when curcumin was used prepartum, nor did the colostrum brix concentration. It was concluded that curcumin supplementation increased serum globulin levels, improving the immune response. Although the curcumin diet was offered only in the prepartum period, there was a positive impact on the immune response of cows for up to seven days postpartum, the most critical moment in dairy cattle farming.

## Simple Summary

The transition period is characterized by profound metabolic and immunological challenges that increase susceptibility to disease in dairy cows. This study evaluated whether prepartum curcumin supplementation could improve health and productive responses during this critical phase. Twenty Holstein cows received curcumin (152 mg/day), while another 20 received a diet without additives (control) during the 21-day prepartum period. Curcumin did not affect milk yield, milk composition, colostrum quality, metabolic biomarkers, or oxidative status. However, cows fed curcumin exhibited higher concentrations of globulin, IgA, immunoglobulin heavy chain, haptoglobin, and transferrin, along with lower levels of C-reactive protein and ceruloplasmin. Prepartum curcumin supplementation enhanced postpartum immune function, supporting its use as a nutritional immunomodulation strategy during the transition period.

## 1. Introduction

The transition period is one of the most critical periods for high-producing cows; the transition period has traditionally been defined as the three weeks before to three weeks after calving [1]. During this time, a cow’s mammary gland remodels and regenerates in preparation for the upcoming lactation. High-producing dairy cows are exposed to stressors such as abrupt cessation of milking, mammary discomfort, and physiological imbalances including altered hormonal, energetic, and immunological status [2,3,4]. Nutrition plays a critical role in the immune response, acting either directly through nutrients or indirectly via biologically active metabolites, particularly during periods of physiological stress [5]. Nutrition plays an essential role in the immune response, and the effect of nutrition can occur directly through nutrients or indirectly through biologically active metabolites, such as in situations of physiological imbalance [5]. Therefore, approximately 21 days before calving, cows need to be separated into management groups according to their metabolic requirements to prepare the cow for a new lactation, avoiding metabolic disorders that can negatively affect the yield, composition, or quality of colostrum and milk [6].

In general, the use of plant-derived phytoactives has resulted in antimicrobial, antioxidant, immunostimulant, and antiviral effects on ruminal fermentation and microbial modulation, improving performance, immunity, and well-being [7,8]. Among the phytoactives that can be used in animal production is curcumin, which is extracted from *Curcuma longa*, popularly called turmeric, a rhizome of the ginger family, used for many centuries by Indians and Chinese in cooking, as a seasoning, dye and for medicinal purposes [9]. Studies have shown that curcumin has anticancer [10,11], anti-inflammatory [12], anti-aging [13], antioxidant [14], cellular radioprotective properties against induced γ radiation [15] and insecticidal [16] properties. In addition, it also prevents inflammation that causes pain by overstimulating pro-inflammatory cytokines, increasing the production of interleukin-10 (IL-10), an anti-inflammatory cytokine [17,18]. In addition, curcumin has been associated with inhibition of cyclooxygenase-2 (COX-2), resulting in reduced production of inflammatory mediators and, consequently, a typical anti-inflammatory effect [19]. Curcumin has already been used in animal production as an additive in some ruminant species [20]. In cattle, the use of a topical herbal formulation with antibacterial and immunomodulatory properties has been reported as an alternative approach for bovine subclinical mastitis [21]. In calves, when curcumin was added to their feed, an increase in circulating antioxidants was observed, as well as a decrease in *Eimeria* infection and increase in weight gain [22]. In a study carried out in lactating sheep, curcumin used as an additive in the diet had an antioxidant and anti-inflammatory effect, increased milk production, decreased somatic cell count (SCC) and protein oxidation [23].

However, the potential use of curcumin as an additive for dairy cows in the transition period is still unknown. Due to the various biological properties of curcumin, our objective was to evaluate whether the addition of curcumin to the diet of prepartum cows would have positive effects on the animals’ productive performance over 180 days postpartum, as well as on the quality of colostrum and milk, as well as on the cows’ health regarding metabolism, immune and oxidative response in the first 28 days of lactation.

## 2. Materials and Methods

The experiment was conducted on a commercial dairy farm (Lacteos Vacaria), located in the municipality of Vacaria, in the Rio Grande do Sul State, Brazil (latitude 28°23′44″, longitude 50°52′31″, and altitude of 914 m above sea level).

### 2.1. Additive: Curcumin

The curcumin used in this research was derived from the dry extract of *Curcuma longa* (turmeric), acquired from Shaanxi Jiahe Phytochem Ltd. (Xi’an, China), with a guaranteed purity greater than 65%. A previously published study by our research group analyzed the purity of curcumin in the product using high-performance liquid chromatography (HPLC) and verified a purity of 71.7% [20].

### 2.2. Animals, Treatment and Experimental Design

The experiment was carried out between February and June (Brazilian autumn) and was approved by the Animal Use Ethics Committee of the Santa Catarina State University (CEUA/UDESC), under protocol number CEUA 9024181225 (ID 002288). The animals were housed in a confined system compost barn, in a closed-sided facility with natural and mechanical ventilation. Environmental conditions were controlled using a water sprinkling system and artificial lighting to maintain thermal comfort and a standardized photoperiod. Feed and water were provided ad libitum through linear feeders and automatic drinkers, respectively.

The experiment was conducted as a continuous trial, with 40 multiparous Holstein cows, grouped into two groups based on milk production in the previous lactation (33.67 ± 9.32 kg/day), and parity (2.87 ± 0.87). Parity was included as a fixed effect in the statistical model to account for its potential confounding influence on metabolic, immunological, and productive responses. Each group was randomly assigned to two treatments, with one group receiving a diet without curcumin (control group) and one group receiving the same diet with the daily addition of 152 mg of curcumin inside capsules (treatment group), as detailed below. Since there were no published articles on the amount of curcumin that could be included in the diet of cows, a pilot study was conducted. In this study, we found that a dose of 152 mg/cow/day is enough to modulate the inflammatory and oxidative response, the focus of our research when using curcumin (unpublished data). Furthermore, this dose of curcumin reduces production costs, since the cost of concentrated curcumin (higher purity) is still high.

All cows enrolled in the experiment were clinically healthy at the beginning of the prepartum period, with no recorded history of metabolic or infectious diseases in the previous lactation, according to farm health records. This inclusion criterion was adopted to minimize the influence of pre-existing health conditions on postpartum metabolic and immune responses.

At that time, we worked with 40 cows, corresponding to a period of 49 days per cow, i.e., 21 days prepartum and 28 days postpartum, to evaluate the effect of the treatment on milk quality and blood parameters. As not all cows were inseminated on the same date, the pre-calving period occurred at two different times, resulting in a 23-day interval between calvings. Milk production was obtained daily between days 5 to 180 of lactation, using the zootechnical control software of the farm’s automatic milking system (AMS). Production data from days prior to day 5 were not registered by the software. The official experimental period was up to 28 days postpartum; however, since we had milk production data due to the robotic system, we chose to include this data in our study.

Cows were confined in a compost barn three weeks before the expected calving date as described above. Both groups were group-fed and received the same pre-calving diet, consisting of concentrate (2.250 kg/day), whole-plant corn silage (24.0 kg/day), and oat straw (3.5 kg/day), provided as a totally mixed diet (TMR) once a day, at 10 A.M. Before feeding the diet, the leftovers in the trough were cleaned every day.

Feed samples (silage, wheat straw, concentrate, and TMR) were collected weekly and dried in a forced-air oven for 3 days (56 °C). At the end of the experiment, all collected samples were combined into a single pooled sample for analysis at a commercial laboratory. The chemical composition of the feed ingredients and total mixed ration (TMR) was determined according to standard analytical procedures. Dry matter (DM), crude protein (CP), ether extract (EE), and ash were analyzed according to AOAC [24], whereas neutral detergent fiber (NDF) was determined according to Van Soest et al. [25]. Results are shown in Table 1.

During the pre-calving period, since the study was conducted on a commercial farm that made it difficult to separate them into groups or control their feeding individually, we chose to have the cows in the treatment group receive curcumin capsules (99% purity), containing 152 mg, once a day. To supply the capsules, the cows were restrained in headlocks; the capsules were placed as a top dressing on a small amount of TMR, and their consumption was monitored individually by the researchers, ensuring curcumin was consumed at its full titer. The animals in the control group received capsules without curcumin (empty capsules). We used gelatin capsules (18 mm size; capacity of 150 to 250 mg), considering their rapid disintegration upon hydration. According to the Brazilian Pharmacopeia, hard gelatin capsules are expected to completely disintegrate within 45 min under the prescribed test conditions [26]. During the experiment, feed samples were collected for bromatological analysis. After calving, the cows were moved to another compost barn system, with access to a free-flow robotic milking system (Lely Astronaut^®^). All animals had access to milking voluntarily since the cows had free access to the robot. Both groups received the same postpartum diet, consisting of concentrate provided when accessing the robot and a total mixed diet ad libitum (Table 1).

### 2.3. Production Control, and Sample Collection

After each animal gave birth, milking was performed as quickly as possible, and a homogeneous sample of colostrum from the four udder quarters was collected to estimate the total solids concentration at room temperature using an optical refractometer. Colostrum quality was assessed based on total solids content and estimated immunoglobulin concentration. The brix percentage is a measure of the sucrose concentration in liquids that do not contain sucrose. There is a high correlation between the brix percentage and the total solids content of the liquid. In dairy science, this measurement is widely used as an indirect and practical indicator of colostrum immunoglobulin G (IgG) concentration. The brix percentage can be correlated with the IgG concentration of the colostrum, and the limit value indicating that the colostrum is of high quality (>50 mg of Ig/mL) is 21% brix, according to the methodology described by Bielmann et al. [27]. The metabolic health of the cow also affects colostrum quality measured as Brix % and may cause failure of passive immunoglobulin transfer as well as impaired calf health [28].

Thus, colostrum samples with Brix values ≥21% were classified as having adequate immunological quality. In addition, a homogeneous 2 mL aliquot of colostrum was collected into sterile tubes for subsequent determination of oxidative biomarkers, which were evaluated as complementary indicators of the oxidative status of colostrum. After calving, cows had free access to an automatic milking system (AMS; Lely Astronaut A4 and A5). Cows were encouraged to visit the milking unit by the availability of concentrate, according to the previously programmed feeding curve. Milk production was automatically recorded at each milking and summarized on a daily basis.

In the first four weeks postpartum (1 to 28 days), a milk sample from the first-morning milking of each animal was collected at 7-day intervals. Milk was collected from all mammary quarters together, using a Lely Shuttle^®^ automatic milk sample collector (Lely, Maassluis, The Netherlands), obtaining an individual sample per cow. Milk quality was evaluated based on its chemical composition, somatic cell count, and physicochemical characteristics. Milk was stored in two standard collection bottles, one of which contained Bronopol^®^ preservative (2-bromo-2-nitropropane-1,3-diol), for analysis of composition (fat, protein, casein, lactose, defatted dry extract and milk urea nitrogen), and SCC, stored at 4 °C until analysis and other, without preservatives performed immediately after collection, was used for physical analyses of the milk (pH, titratable acidity, alcohol test, cryoscopy).

On the day of calving (day 0) and days 7, 14, 21 and 28 postpartum, blood parameters were collected by coccygeal venipuncture in vacuum tubes, blood samples were collected in the morning, before feeding and milking, to minimize metabolic variation. The samples were kept refrigerated and centrifuged at 3000 rpm for 15 min at 4 °C to obtain serum, which was identified and frozen at −20 °C until the time of analysis. Blood serum samples were used for biochemical parameters, Ferric reducing antioxidant power (FRAP), glutathione S-transferase (GST), and superoxide dismutase (SOD) analyses, and whole blood was used for BHBA determination.

### 2.4. Composition and Physicochemical Properties of Milk

The fat, protein, lactose, casein and milk urea nitrogen (MUN) contents of the collected milk were determined using the infrared method (Bentley NexGen—Bentley Instruments^®^, Bentley Instruments, Chaska, MN, USA) and the somatic cell count (SCC) was measured using flow cytometry (Bentley NexGen—Bentley Instruments^®^). For the alcohol resistance test, 2 mL of milk and 2 mL of alcohol were added to a Petri dish on a black background and homogenized, considering the sample stable at the alcohol concentration prior to the formation of lumps. The alcohol concentrations tested were 56 to 86% *v*/*v*, with 2% intervals. The titratable acidity was determined in Dornic degrees (°D), by transferring 10 mL of the milk sample to a beaker; then, 3 drops of 1% phenolphthalein were added and titrated with Dornic solution (0.11 N or N/9) until a persistent pink color appeared for approximately 30 s.

### 2.5. Serum Biochemistry

Serum levels of total proteins and albumin, as well as activities of alanine aminotransferase (ALT), gamma glutamyl transferase (GGT) and aspartate aminotransferase (AST) were assessed using commercial kits (Gold Analisa Diagnóstica Ltd.a^®^, Carlos Prates, Belo Horizonte, MG, Brazil), in semi-automatic equipment (Bio Plus 2000^®^, São Paulo, Brazil), in the laboratory of the State University of Santa Catarina. Globulin levels were calculated by subtracting albumin levels from total protein levels.

After blood collections during the experiment, peripheral blood parameters were collected to determine the concentration of beta-hydroxybutyrate (BHBA). The analyses were performed using a portable Optium Xceed analyzer (Abbott, Chicago, IL, USA). Beta-hydroxybutyrate analysis strips (Abbott, Chicago, IL, USA) were used.

### 2.6. Oxidants and Antioxidants

Lipid peroxidation was assessed by measuring thiobarbituric acid reactive substances (TBARS) according to the method described by Jentzsch et al. [29]. Absorbance was read at 535 nm using a spectrophotometer (Shimadzu, Kyoto, Japan), and results were expressed as nmol of malondialdehyde per mL of serum.

The total antioxidant potential of the different samples was determined using the method described by Benzie and Strain [30], through the ferric ion reducing antioxidant power (FRAP). A calibration curve [FRAP = 1000 × absorbance; R2 = 0.9936] was obtained, using different concentrations of trolox (100–1000 μmol/L). The results were expressed as μmol of trolox equivalents per liter of smoothie (μmoL TE/L), using a microplate reader (Epoch microplate spectrophotometer, Synergy-BioTek, Winooski, VT, USA).

GST activity was measured according to Mannervik and Guthenberg [31], with minor modifications described in sequence. The activity was measured by the rate of formation of dinitrophenyl-S-glutathione at 340 nm in a medium containing 50 mM potassium phosphate, pH 6.5, 1 mM GSH, 1 mM 1-chloro-2,4-dinitrobenzene (CDNB) as substrate and tissue supernatants (approximately 0.045 mg protein). The results were calculated and expressed as U/mg protein.

SOD activity in serum were evaluated spectrophotometrically, as described by Marklund and Marklund [32], based on the reaction of inhibition of superoxide anion in the presence of pyragolol. Enzymatic activity was expressed in SOD U/mg protein.

### 2.7. Proteinogram

For protein fractionation, sodium dodecyl sulfate-polyacrylamide gel electrophoresis (SDS-PAGE) was performed according to the technique suggested by Fagliari et al. [33] adapted by Tomasi et al. [34], using a mini gel (10 × 10 cm). The gel was stained with Comassie Blue and photographed to identify and quantify the protein fractions using the Labimage1D software (Version: 7.4.2, Loccus Biotechnology, São Paulo, SP, Brazil). A standard containing fractions with molecular weight between 10 and 250 kDa (Kaleidoscope—Biorad) was used as a reference to identify the protein fractions.

### 2.8. Statistical Analysis

Sample size was determined following the approach described by Dohoo et al. [35] for comparison of two means, using the following equation:

(1) $$n=\frac{2\sigma^{2}{\left(Z_{1}-\frac{\alpha }{2}+Z_{1}-\beta \right)}^{2}}{d^{2}}$$

where *n* is the number of animals per group, “*σ*^2^” is the variance of milk yield, “*d*” is the minimum detectable difference between treatments, “*Z*_1_ − *α*/2” is the standard normal deviate corresponding to a two-sided significance level of 5% (1.96), and “*Z*_1_ − *β*” corresponds to the desired statistical power. Assuming a two-sided *α* = 0.05, 90% statistical power (*Z*_1_ − *β* = 1.28), a standard deviation of 5 kg/day for milk yield during early lactation, and a minimum expected difference of 3 kg/day between treatments, a sample size of 20 cows per treatment was considered adequate to detect the expected treatment effect. The repeated-measures design, with multiple observations collected from each cow throughout the postpartum period, was incorporated into the experimental design to account for within-animal variation over time and improve the efficiency of treatment comparisons. Similar sample sizes (*n* = 20 cows per treatment) have been used in previous studies evaluating nutritional interventions in postpartum dairy cows [36,37].

Statistical analysis was performed with SAS 9.4 software (SAS Institute Inc., Cary, NC, USA). The normality of the data was tested via the Kolmogorov–Smirnov test with a significance level of 0.05. ANOVA was performed using the MIXED procedure with repeated measures over time, with significance set at *p* < 0.05 and a tendency at *p* ≤ 0.10.

The statistical model includes the effects of treatments (curcumin or control), parity (two or three or more calvings), week of sample collection, the interactions treatments × parity, treatments × week, parity × week, treatments × parity × week, and the experimental error. For the variables related to colostrum, the model consisted only of the variables’ treatments, parity treatments ×x parity, and the experimental error.

For milk production, daily milk yield records from robotic milking during the first 6 months of lactation for each cow were used, with the average monthly production serving as the experimental unit. The statistical model included the effects of treatments, parity, month of lactation, treatments × parity, treatments × month, parity × month, treatments × parity × month, and the experimental error.

## 3. Results

### 3.1. Milk Production and Quality

No differences were detected between treatments, nor was there any interaction between treatment and lactation month for milk production in the first 6 months of lactation (Table 2). The addition of curcumin in the prepartum period also did not influence milk composition during lactation regarding fat, protein, casein, lactose, fat: protein ratio, casein: protein ratio, total dry extract, defatted dry extract and urea nitrogen in milk (*p* ≥ 0.05). Effect of the week of lactation (*p* = 0.04) was found in the fat, protein, casein, lactose, defatted dry extract, and total dry extract. However, no interaction was observed between the week and treatment (*p* = 0.10) (Table 2).

The physicochemical properties of milk (cryoscopy, pH and resistance to the alcohol test) were not affected by the addition of curcumin to the diet of cows in the prepartum period, as there was no effect of treatment (*p* = 0.4201) and no interaction between treatment and week (*p* = 0.668). However, there was an effect of the week for the variables pH, resistance to the alcohol test and titratable acidity (*p* = 0.0008) (Table 2).

### 3.2. Serum Biochemistry

There was an interaction between treatment × week (*p* = 0.005) and week of collection (*p* < 0.001) for serum globulin concentrations (Table 3). Cows treated with curcumin had higher globulin concentrations on the seventh day postpartum. No effect of treatment was observed, nor was there any effect of the treatment × day interaction for serum concentrations of ALT, GGT, AST, albumin, beta-hydroxybutyrate and total protein; however, there was an effect of collection day for the variables AST, albumin and beta-hydroxybutyrate (Table 3).

### 3.3. Proteinogram

There was an effect of treatment (*p* ≤ 0.001, Table 4) and collection day (*p* = 0.017) for the variable’s IgA and heavy chain Ig, which were higher in animals that consumed curcumin. The variables haptoglobin and transferrin showed treatment effect (*p* = 0.032), treatment × day interaction (*p* = 0.028), and day effect (*p* = 0.0024), with higher means for the curcumin group. Serum levels of C-reactive protein and ceruloplasmin were lower in the blood of cows fed curcumin compared to control cows (*p* = 0.0002), also showing a day effect for C-reactive protein (*p* ≤ 0.0001).

### 3.4. Serum Oxidant/Antioxidant Status

No treatment effect (*p* = 0.189) or treatment × day interaction (*p* = 0.468) were detected for serum concentrations of FRAP and TBARS, or for GST and SOD activity in the serum blood. The FRAP and SOD variables showed a day effect (*p* = 0.01), being higher on day 7 in both groups (Table 5).

### 3.5. Colostrum Quality

No differences were detected between treatments and treatment × parity interaction (*p* ≥ 0.10) for the variables of brix, total protein and FRAP levels and GST activity in colostrum (Table 6). There was, however, a trend of treatment effect (*p* = 0.07) for SOD activity, with cows fed curcumin tending to have colostrum with higher levels of this antioxidant enzyme compared to the control group.

## 4. Discussion

This study analyzed the effect of adding curcumin to the diet of prepartum cows on variables analyzed postpartum, i.e., the evaluation was based on indirect effects at the beginning of the animals’ lactation. The addition of curcumin prepartum did not influence milk production. However, it is important to emphasize that the experiment’s animals have high genetic and production levels, reaching an average of 50.31 kg/day. Marcon et al. [38] observed greater average daily weight gain in lambs supplemented with curcumin without increasing feed consumption. Glombowsky et al. [22] also reported that calves fed curcumin had greater weight gain. Furthermore, in a study carried out with curcumin in the diets of lactating sheep, it was found that the use of this additive had beneficial effects on the health, and performance of the animals, since it increased the capacity for absorption of nutrients in the gastrointestinal tract and, consequently, determined greater growth and modulation of the antioxidant and immune system, reducing the activity of pathogenic organisms [23]. Evidence in the literature suggests that prepartum feeding strategies that incorporate nutraceuticals can improve the immune status and metabolic activity of central organs involved in nutrient utilization and milk synthesis, such as the intestine, mammary gland, and liver [7]. The results of this study do not indicate significant differences in the composition and quality of milk, but it influenced the immune response.

The postpartum period is characterized by high energy demand, to which the cow adapts metabolically by mobilizing non-esterified fatty acids (NEFA), also increasing beta-hydroxybutyrate (BHB), to meet the demands of lactation [39]. Therefore, BHB has been reported as a good marker for postpartum energy metabolism [40]. According to the reference range described by González et al. [41], the postpartum BHB concentrations observed in this study remained within normal limits, corroborating the interpretation that the cows underwent a successful transition period, regardless of curcumin intake. In addition, the group of animals that received curcumin had higher serum concentrations of haptoglobin than those in the control group. Although an increase in haptoglobin was observed, the concentrations remained within the physiological reference range described for dairy cows in the postpartum period [41,42], indicating that this response does not characterize a pathological inflammatory condition. According to Bhat et al. [43], negative energy balance in cows is usually related to increased haptoglobin levels. However, as non-esterified fatty acids (NEFA) were not measured in the present study, it is not possible to establish a direct association between haptoglobin concentrations and energy balance. Haptoglobin is considered one of the main positive acute phase proteins produced by the liver, which increases considerably with liver injury or overload and binds to free hemoglobin in the blood to conserve Fe and prevent it from becoming free in the blood, where it would be available to potential pathogenic microbes [44]. Considering that indicators of energy metabolism and liver function remained within normal limits [41,45], the increase in haptoglobin may reflect a regulated acute phase response rather than metabolic stress, although this response should be further investigated in future studies.

A positive effect of adding curcumin is related to globulin levels (Table 4), as demonstrated by the fact that the cow group that received curcumin showed higher values on the seventh day of lactation. Molosse et al. [46] observed lower protein and globulin levels in lambs fed curcumin. According to Gonzalez [47], during the transition period, decreased serum protein levels are associated with lower immunity, and reduced total protein levels are associated with reduced globulin levels, which are important proteins for the development of an effective immune response [48]. In the present study, the increase in globulin levels was interpreted as a positive effect, as globulins comprise immunologically active proteins, including immunoglobulins, which play a central role in humoral immune responses and are essential for protection against pathogens during the early postpartum period. Thus, higher globulin concentrations suggest improved immune competence in cows during the first days of lactation. The increase in globulins was reflected in higher concentrations of heavy chain IgG and IgA in animals that received curcumin (Table 5). According to 22. Glombowsky et al. [22], curcumin stimulated an increase in immunoglobulin levels in the blood of calves. These findings support the hypothesis that curcumin may act as an immunomodulator in ruminants, although the magnitude of this effect likely depends on factors such as dosage, formulation, rumen stability, and bioavailability. Thus, adding curcumin to the diet can prevent immune system impairment [49]. A study by Santos et al. [50,51] demonstrated that the use of herbal medicine in cows’ diets in the prepartum period reflected the improvement in the animals’ immune response, although the authors emphasize that the magnitude of productive responses may vary depending on experimental conditions, and in the present study no significant effects were observed on milk production or SCC.

The higher serum transferrin levels seven days postpartum in cows supplemented with curcumin in the prepartum period may be related to the anti-inflammatory effects of curcumin [52]. Transferrin is a negative acute phase protein in cows, i.e., its concentration decreases during inflammatory events, such as calving or other events common in the transition period, such as metritis, mastitis, placental retention and ketosis [45]. In the present study, transferrin concentrations remained within physiological limits [42,45], and the observed increase suggests a lower inflammatory challenge in cows fed with curcumin, reinforcing the beneficial effect and its inclusion in prepartum.

The evaluation of the metabolic profile of dairy cows is very important for the early diagnosis, treatment and prevention of production diseases in the transition period since the negative consequences during prepartum can influence production, especially at the beginning of lactation [53,54]. The lower serum levels of ceruloplasmin and C-reactive protein up to seven days after parturition in cows fed curcumin (Table 5) indicate a positive result for curcumin intake since ceruloplasmin and C-reactive protein can be used as immunological indicators, but some authors mention that they would be indicators of animal well-being and health, since higher levels of these proteins are reported in cows with higher stress levels and poor health and management conditions [55,56]. Importantly, the concentrations of ceruloplasmin and C-reactive protein observed in both experimental groups remained within physiological reference ranges [42,55], indicating modulation of the acute phase response rather than pathological inflammation. For clarity, the concentrations of acute phase proteins evaluated in this study are expressed in their respective units of measurement (mg/dL or g/L, as indicated in Table 4 and Table 5), allowing adequate interpretation of the results and comparison with reference values reported in the literature. The transition period is a time of immunosuppression, characterized by a marked decrease in the responsiveness to injury and infectious challenge. Therefore, as ceruloplasmin and C-reactive protein constitute acute phase proteins in cows, an increase may occur due to the intensity of physiological stress and pathological events in this period [42,56,57]. Furthermore, ceruloplasmin and C-reactive protein are used as an important source of information for the detection of mastitis in cows [57,58]. Kaya et al. [59] also found that serum ceruloplasmin levels were higher in cows with endometritis than in healthy cows and that the severity of endometritis increases considerably when serum ceruloplasmin levels are higher. Thus, the reduction in these biomarkers in cows fed with curcumin suggests a biologically relevant anti-inflammatory effect, even though values remained within normal physiological limits [42].

Curcumin is known for its potential antioxidant effect due to its ability to sequester free radicals in the body, such as reactive oxygen species, and donate hydrogen electrons, which allows cell stability, inhibiting the cascade effect of oxidative stress. This is a consequence of the imbalance between the concentration of free radicals and the antioxidant system [60]. Adding curcumin to the diet of lactating sheep increased the levels of total antioxidants and decreased the levels of oxidants in the milk of animals that received the additive [23]. Although curcumin is known for this effect, in the present study, there was no difference in the blood and colostrum of the animals for oxidants and antioxidants (Table 6), but rather a tendency for greater SOD activity in the colostrum. Since curcumin is a molecule with a short half-life in the animal’s body, the non-consumption of this additive during lactation reflects the absence of antioxidant activity in the blood and colostrum of the cows, but we cannot rule out the lack of effect being directly related to the high productivity of the cows, which are under constant challenge, just as the daily dose of curcumin may have been low. Overall, although the observed differences in acute phase proteins did not exceed physiological thresholds, the consistent pattern of reduced positive acute phase proteins and increased negative acute phase proteins and immunoglobulins indicates biologically relevant modulation of the immune and inflammatory response in early lactation [7,42].

The significant effect of parity observed for TBARS concentrations suggests that oxidative status of colostrum may be influenced by the physiological and metabolic differences associated with lactation number. Previous research has demonstrated differences in the antioxidant characteristics of colostrum according to parity, with colostrum from primiparous Holstein cows showing greater antioxidant capacity than that from multiparous cows, including higher oxygen radical absorbance capacity and ceruloplasmin activity [61]. Similarly, studies evaluating systemic oxidative status in dairy cows have reported parity-related differences in pro- and antioxidant biomarkers during the transition period, indicating that cows in different lactations may respond differently to the metabolic demands associated with calving and the onset of lactation [62,63]. In the present study, the parity effect on TBARS, independent of treatment and without a treatment × parity interaction, suggests that differences in lipid peroxidation may be related to the physiological stage and metabolic history associated with parity rather than to the dietary treatment.

This study was conducted on a dairy farm using diets and management practices typical of high-producing cows in a robotic milking system. Such conditions limit the assessment of individual feed intake; consequently, further studies at research centers are needed to better understand the effects of including curcumin in prepartum dairy cow diets on subsequent productive performance. It is well known that feed intake influences metabolic, productive, and milk composition variables; therefore, we emphasize the need for caution when interpreting these data, which did not differ statistically. However, the key immunological and oxidative effects observed in our study likely stem from the addition of curcumin to the diet rather than from dry matter intake, given that both experimental groups received the same diet during the pre- and post-partum periods.

## 5. Conclusions

In the conditions of this study, the addition of curcumin to the diet of dairy cows in the prepartum period did not affect milk production and composition in the subsequent lactation. However, it was able to improve the immunity of cows in the postpartum period, with an increase in globulin levels due to the increase in IgA immunoglobulins and heavy chain immunoglobulins combined with a reduction in positive acute phase proteins (C-reactive protein and ceruloplasmin), and an increase in negative acute phase proteins (transferrin), which suggests an anti-inflammatory effect that is desired in production animals. Although curcumin is considered an antioxidant additive, the investigated biomarkers did not differ between the experimental groups. This is the first study on curcumin addition for high-yield confined cows during the prepartum period; further studies are needed to evaluate the feasibility of this supplementation as a dietary strategy for dairy herds.

## Figures and Tables

**Table 1:** Chemical composition of the ingredients used in the diet prepartum and postpartum.

Chemical Composition	Silage	Wheat Straw	Concentrate ^1^	Concentrate ^2^	TMR ^3^	TMR ^4^
Dry matter (DM), %	36.8	82.2	93.26	95.90	77.06	84.93
Ash % DM	4.10	NE	32.83	43.48	40.72	32.24
Crude protein, % DM	10.6	5.57	34.39	16.73	12.31	16.93
NDF, % DM	41.0	72.6	32.65	40.65	46.09	30.67

Concentrate ^1^ prepartum; Concentrate ^2^ robot; TMR ^3^—total prepartum mixed ration. TMR ^4^—total postpartum mixed ration. NE—(not-evaluated).

**Table 2:** Means, standard error of the mean (SEM) and *p* values for milk production (5 to 180 days in lactation) and composition (1, 7, 14, 21 and 28 days in lactation) of cows fed diets with and without curcumin in prepartum.

Variables	Treatment ^1^		SEM	*p*-Value		
	Control	Curcumin		Treatment	Week	Treat × Time
Milk production (kg/day)	49.61	50.84	2.87	0.7626	<0.0001	0.484
Fat (%)	3.58	3.56	0.13	0.9161	0.0046	0.978
Protein (%)	3.27	3.13	0.05	0.1084	<0.0001	0.929
Fat: protein relation (–)	1.10	1.13	0.25	0.5136	0.920	0.990
Casein (%)	2.60	2.49	0.05	0.1283	0.0001	0.762
Casein: protein relation (–)	0.79	0.79	0.00	0.8969	0.040	0.268
Lactose (%)	4.61	4.64	0.03	0.5903	0.001	0.188
Defatted Dry Extract (%)	8.81	8.69	0.07	0.2885	0.001	0.676
Total Dry Extract (%)	12.38	12.25	0.17	0.5890	0.0001	0.982
MUN ^1^ (mg/dL)	13.8	13.0	0.54	0.3464	0.420	0.502
SCC ^2^ (cells/mL)	1.73	2.54	0.44	0.2067	0.510	0.145
Titratable acidity (ºD)	19.17	18.49	0.59	0.4201	0.0001	0.915
Alcohol Test (%)	68.45	69.30	0.86	0.4935	0.0004	0.747
pH (–)	6.72	6.71	0.01	0.4589	0.0008	0.975
Cryoscopy (°H)	−0.5327	−0.5301	0.00	0.4997	0.9937	0.668

^1^ Milk Urea Nitrogen; ^2^ Somatic Cell Count.

**Table 3:** Means, standard error of the mean (SEM) and *p* values for serum biochemical variables of cows fed diets with and without curcumin in the prepartum period: evaluation performed up to 28 days postpartum.

Variables	Treatment ^2^		SEM	*p*-Value		
	Control	Curcumin		Treatment	Day	Treatment × Week
ALT ^1^ (U/L)	14.57	13.72	0.88	0.317	0.25	0.910
GGT (U/L)	31.1	31.1	4.39	0.993	0.09	0.726
AST (U/L)	49.61	52.52	1.81	0.262	0.001	0.347
Total protein (g/dL)	6.58	6.68	0.09	0.119	0.1468	0.516
Albumin (g/dL)	3.05	2.98	0.06	0.474	0.001	0.330
Globulin (g/dL)	3.66	3.84	0.09	0.352	0.0001	0.005
Beta-hydroxybutyrate (mmol/L)	0.66	0.67	0.04	0.7842	<0.001	0.647

^1^ Alanine aminotransferase (ALT), Gamma Glutamyl Transferase (GGT), Aspartate aminotransferase (AST); ^2^ The CURCUMIN and CONTROL treatments represent a diet with 152 mg/day of curcumin or a diet without curcumin, respectively.

**Table 4:** Means, standard error of the mean (SEM) and *p* values of serum protein concentration of cows fed diets with and without curcumin in the prepartum period: evaluation performed days 0 (partum), 7 and 21 days postpartum.

Variables	Treatment ^2^		SEM	*p*-Value		
	Control	Curcumin		Treatment ^3^	Day	Treatment × Day ^4^
IgA (g/dL) ^1^	0.66 ^b^	0.76 ^a^	0.01	0.001	0.001	0.396
D0	0.61	0.69	0.02			
D7	0.72	0.84	0.02			
D21	0.67	0.75	0.02			
IG heavy chain (g/dL) ^1^	0.82 ^b^	0.92 ^a^	0.01	0.0005	0.017	0.394
D0	0.80	0.88	0.02			
D7	0.85	0.97	0.02			
D21	0.83	0.94	0.02			
Ceruloplasmin (g/dL)	0.61 ^a^	0.48 ^b^	0.02	0.0002	0.71	0.259
D0	0.62	0.48	0.02			
D7	0.59	0.49	0.02			
D21	0.60	0.49	0.02			
C-reactive protein (g/dL)	0.33 ^a^	0.24 ^b^	0.01	<0.0001	<0.0001	0.151
D0	0.43	0.33	0.01			
D7	0.23	0.16	0.01			
D21	0.32	0.25	0.01			
Haptoglobin (g/dL)	0.24	0.34	0.01	<0.0001	<0.0001	0.028
D0	0.25 ^B^	0.43 ^A^	0.01			
D7	0.22	0.25	0.01			
D21	0.25	0.33	0.02			
Transferrin (g/dL)	0.22 ^b^	0.26 ^a^	0.01	0.032	0.0024	0.006
D0	0.22	0.21	0.01			
D7	0.23 ^B^	0.31 ^A^	0.01			
D21	0.23	0.27	0.01			

^1^ Immunoglobulin A (IgA), Immunoglobulin heavy chain (IG heavy chain). ^2^ CURCUMIN and CONTROL treatments represent a diet with 152 mg/day of turmeric or a diet without turmeric, respectively. ^3^ Treatment effect (*p* < 0.05) is illustrated by different lowercase letters (^a,b^) on the same line. ^4^ Treatment × day interaction (*p* < 0.05) is illustrated by different uppercase letters (^A,B^) on the same line of the collection period.

**Table 5:** Serum levels of oxidants/antioxidants of cows fed a diet with and without curcumin in the prepartum period: evaluation performed days 0 (partum), 7 and 21 days postpartum.

Variables ^1^	Treatment ^2^		SEM	*p*-Value		
	Control	Curcumin		Treatment ^4^	Day ^3^	Treatment × Day ^4^
FRAP (μmol/L)	1.08	1.13	0.05	0.4971	0.01	0.873
D0	1.06 ^B^	1.10 ^B^	0.06			
D7	1.18 ^A^	1.21 ^A^	0.06			
D21	1.01 ^B^	1.09 ^B^	0.06			
GST (U GST/mg protein)	7.43	6.56		0.1891	0.98	0.468
D0	7.60	6.52	0.64			
D7	8.06	5.88	0.64			
D21	6.63	7.29	0.64			
SOD (U SOD/mg protein)	2.84	2.71	0.18	0.6260	0.002	0.615
D0	3.10 ^A^	3.23 ^A^	0.25			
D7	2.49 ^B^	2.27 ^B^	0.25			
D21	2.29 ^B^	2.29 ^B^	0.25			
TBARS (nmol/mL)	5.70	5.12	0.31	0.2112	0.290	0.113
D0	5.27	4.79	0.47			
D7	6.46	4.94	0.47			
D21	5.36	5.65	0.47			

^1^ Ferric reducing capacity of plasma (FRAP), glutathione S-transferase (GST), superoxide dismutase (SOD), and thiobarbituric acid reactive species (TBARS). ^2^ CURCUMIN and CONTROL treatments represent a diet with 152 mg/day of turmeric or a diet without turmeric, respectively. ^3^ The effect of the day on each treatment was illustrated by different capital letters (^A,B^) in the same column for each variable. ^4^ There was no treatment effect or treatment × day interaction (*p* < 0.05) for oxidative status biomarkers.

**Table 6:** Brix and oxidant/antioxidant levels in the colostrum of cows fed a diet with and without curcumin prepartum.

Variables ^1^	Treatment ^2^		SEM	*p*-Value		
	Control	Curcumin		Treatment	Parity	Treatment × PO
Protein (mg/mL)	4.60	4.07	0.46	0.4404	0.69	0.816
FRAP (µmol/L)	0.84	0.85	0.03	0.6455	0.51	0.314
GST (U mg/protein)	10.3	12.0	2.17	0.5544	0.77	0.774
SOD (U mg/protein)	2.04 ^b^	3.45 ^a^	0.52	0.0746	0.75	0.416
TBARS (nmol MDA/mL)	5.52	6.44	0.63	0.1428	0.01	0.981
BRIX	26.6	27.1	0.88	0.7206	0.27	0.446

^1^ Ferric reducing capacity of plasma (FRAP), glutathione S-transferase (GST), superoxide dismutase (SOD), and thiobarbituric acid reactive species (TBARS). ^2^ CURCUMIN and CONTROL treatments represent a diet with 152 mg/day of turmeric or a diet without turmeric, respectively. Values within the same row with different superscript letters (^a,b^) differ significantly (*p* < 0.05).

## Data Availability

The data presented in this study are available on request from the corresponding authors.

## References

[1] Grummer R.R. (1995). Impact of changes in organic nutrient metabolism on feeding the transition dairy cow. J. Anim. Sci..

[2] Batistel F., Arroyo J.M., Garces C.I.M., Trevisi E., Parys C., Ballou M.A., Loor J.J. (2018). Ethyl-cellulose rumen-protected methionine alleviates inflammation and oxidative stress and improves neutrophil function during the periparturient period and early lactation in Holstein dairy cows. J. Dairy Sci..

[3] Mccabe C.J., Boerman J.P. (2020). Revisão convidada: Quantificação da mobilização de proteínas em vacas leiteiras durante o período de transição. Appl. Anim. Sci..

[4] Zobel G., Weary D.M., Leslie K.E., Von Keyserlingk M.A.G. (2015). Invited review: Cessation of lactation: Effects on animal welfare. J. Dairy Sci..

[5] Ingvartsen K.L., Moyes K. (2013). Nutrition, immune function and health of dairy cattle. Animal.

[6] Andrew S., Otterby D.E. (2001). Availability, storage, treatment, composition and feeding value of surplus colostrum: A review. J. Dairy Sci. Champaign.

[7] Lopreiato V., Mezzetti M., Cattaneo L., Ferronato G., Minuti A., Trevisi E. (2020). Role of nutraceuticals during the transition period of dairy cows: A review. J. Anim. Sci. Biotechnol..

[8] Trevisi E., Zecconi A., Cogrossi S., Razzuoli E., Grossi P., Amadori M. (2014). Strategies for reduced antibiotic usage in dairy cattle farms. Res. Vet. Sci..

[9] Chaturvedi T.P. (2009). Uses of turmeric in dentistry: An update. Indian J. Dent. Res..

[10] Aggarwal B.B., Kumar A., Bharti A.C. (2003). Anticancer potential of curcumin: Preclinical and clinical studies. Anticancer Res..

[11] Shehzad A., Lee J., Lee Y.S. (2013). Curcumin in various cancers. Biofactors.

[12] Joe B., Vijaykumar M., Lokesh B. (2004). Biological properties of curcumin-cellular and molecular mechanisms of action. Crit. Rev. Food Sci. Nutr..

[13] Lima C.F., Pereira-Wilson C., Rattan S.I. (2011). Curcumin induces heme oxygenase-1 in normal human skin fibroblasts through redox signaling: Relevance for anti-aging intervention. Mol. Nutr. Food Res..

[14] Imlay J.A. (2003). Pathways of oxidative damage. Annu. Rev. Microbiol..

[15] Sebastià N., Montoro A., Montoro A., Almonacid M., Villaescusa J.I., Cervera J., Soriano J.M. (2011). Assessment in vitro of radioprotective efficacy of curcumin and resveratrol. Radiat. Meas..

[16] Amalraj A., Pius A., Gopi S., Gopi S. (2017). Atividades biológicas de curcuminóides, outras biomoléculas de açafrão e seus derivados—Uma revisão. Rev. Med. Tradic. Complement..

[17] Fattori P., Breveglieri R., Bosco A., Gamberini M., Galletti C. (2015). Vision for prehension in the medial parietal cortex. Cereb. Cortex.

[18] Kim D.K., Lillehoj H.S., Lee S.H., Jang S.I., Lillehoj E.P., Bravo D. (2013). Dietary *Curcuma longa* enhances resistance against *Eimeria maxima* and *Eimeria tenella* infections in chickens. Poult. Sci..

[19] Lev-Ari S., Strier L., Kazanov D., Madar-Shapiro L., Dvory-Sobol H., Pinchuk I., Arber N. (2005). Celecoxib and curcumin synergistically inhibit the growth of colorectal cancer cells. Clin. Cancer Res..

[20] Damo M., Wandscheer J.G.W., Signor M.H., Marcon C., Nora L., Xavier A.C.H., Wagner R., Vedovatto M., Silva A.S.D. (2026). Curcumin as a dietary additive in early-finished feedlot steers and its effects on performance, ruminal environment, animal health, and meat quality. Animals.

[21] Bhatt V.D., Shah T.M., Nauriyal D.S., Kunjadia A.P., Joshi C.G. (2014). Evaluation of a topical herbal drug for its in-vivo immunomodulatory effect on cytokines production and antibacterial activity in bovine subclinical mastitis. Ayu.

[22] Glombowsky P., Volpato A., Campigotto G., Soldá N.M., Dos-Santos D.S., Bottari N.B., Da-Silva A.S. (2020). Dietary addition of curcumin favors weight gain and has antioxidant, anti-inflammatory and anticoccidial action in dairy calves. Rev. Colomb. Cienc. Pecu..

[23] Jaguezeski A.M., Perin G., Bottari N.B., Wagner R., Fagundes M.B., Schetinger M.R.C., Da Silva A.S. (2018). Addition of curcumin to the diet of dairy sheep improves health, performance and milk quality. Anim. Feed Sci. Technol..

[24] AOAC (2000). Official Methods of Analysis of AOAC International.

[25] Van Soest P.J., Robertson J.B., Lewis B.A. (1991). Methods for dietary fiber, neutral detergent fiber, and nonstarch polysaccharides in relation to animal nutrition. J. Dairy Sci..

[26] Brazilian Health Regulatory Agency (ANVISA) (2019). Brazilian Pharmacopoeia.

[27] Bielmann V., Gillan J., Perkins N.R., Skidmore A.L., Godden S., Leslie K.E. (2010). An evaluation of Brix refractometry instruments for measurement of colostrum quality in dairy cattle. J. Dairy Sci..

[28] Immler M., Failing K., Gärtner T., Wehrend A., Donat K. (2021). Associations between the metabolic status of the cow and colostrum quality as determined by Brix refractometry. J. Dairy Sci..

[29] Jentzsch A.M., Bachmann H., Furst P., Biesalski H. (1996). Improved analysis of malondialdehyde in human body fluids. Free Radic. Biol. Med..

[30] Benzie I.F., Strain J.J. (1996). The ferric reducing ability of plasma (FRAP) as a measure of “antioxidant power”: The FRAP assay. Anal. Biochem..

[31] Mannervik B., Guthenberg C. (1981). Glutathione transferase (human placenta). Methods Enzymol..

[32] Marklund S., Marklund G. (1974). Involvement of the superoxide anion radical in the autoxidation of pyrogallol and a convenient assay for superoxide dismutase. Eur. J. Biochem..

[33] Fagliari J.J., McClenahan D., Evanson O.A., Weiss D.J. (1998). Changes in plasma protein concentrations in ponies with experimentally induced alimentary laminitis. Am. J. Vet. Res..

[34] Tomasi T., Volpato A., Pereira W.A.B., Debastiani L.H., Bottari N.B., Morsch V.M., Schetinger M.R.C., Leal M.L.R., Machado G., Da Silva A.S. (2018). Metaphylactic effect of minerals on the immune response, biochemical variables and antioxidant status of newborn calves. J. Anim. Physiol. Anim. Nutr..

[35] Dohoo I., Martin W., Stryhn H. (2009). Veterinary Epidemiologic Research.

[36] Lora I., Massignani M., Stefani A., Gottardo F. (2021). Potential Benefits to Dairy Cow Welfare of Using a Ceftiofur-Ketoprofen Combination Drug for the Treatment of Inflammatory Disease Associated with Pyrexia: A Field Clinical Trial on Acute Puerperal Metritis. Animals.

[37] Pirestani A., Motalebipour E.Z. (2025). Variation in Uterine and Serum Proteins During the Reproductive Cycle in Dairy Cows Following Low and High Dietary Protein Intake. Vet. Med. Sci..

[38] Marcon H., Souza C.F., Baldissera M.D., Alba D.F., Favaretto J.A., Santos D.S., Da Silva A.S. (2021). Effect of curcumin dietary supplementation on growth performance, physiology, carcass characteristics and meat quality in lambs. Ann. Anim. Sci..

[39] Piñeiro J.M., Menichetti B.T., Barragan A.A., Relling A.E., Weiss W.P., Bas S., Schuenemann G.M. (2019). Associations of pre-and postpartum lying time with metabolic, inflammation, and health status of lactating dairy cows. J. Dairy Sci..

[40] Mann S., Nydam D.V., Abuelo A., Yepes F.L., Overton T.R., Wakshlag J.J. (2016). Insulin signaling, inflammation, and lipolysis in subcutaneous adipose tissue of transition dairy cows either overfed energy during the prepartum period or fed a controlled-energy diet. J. Dairy Sci..

[41] González F.D., Muiño R., Pereira V., Campos R., Benedito J.L. (2011). Relationship among blood indicators of lipomobilization and hepatic function during early lactation in high-yielding dairy cows. J. Vet. Sci..

[42] Eckersall P.D. (2008). Proteins, proteomics, and the dysproteinemias. Clin. Biochem. Domest. Anim..

[43] Bhat S.V., Anisha J.P., Shynu M., Jayavardhanan K.K., Ramnath V. (2020). Higher concentration of haptoglogin indicates transient inflammation and negative energy balance in transition cows. J. Vet. Anim. Sci..

[44] Petersen H., Nielsen J., Heegaard P.M.H. (2004). Application of acute phase protein measurements in veterinary clinical chemistry. Vet. Res..

[45] Kaneko J.J., Harvey J.W., Bruss M.L. (2008). Clinical Biochemistry of Domestic Animals.

[46] Molosse V., Souza C.F., Baldissera M.D., Glombowsky P., Campigotto G., Cazaratto C.J., da Silva A.S. (2019). Diet supplemented with curcumin for nursing lambs improves animal growth, energetic metabolism, and performance of the antioxidant and immune systems. Small Rumin. Res..

[47] Gonzalez D.D., Santos M.J.D. (2017). Bovine colostral cells—The often forgotten component of colostrum. J. Am. Vet. Med. Assoc..

[48] Fernández-Cruz E., Alecsandru D., Ramón S.S. (2009). Mechanisms of action of immune globulin. Clin. Exp. Immunol..

[49] Mallo N., DeFelipe A.P., Folgueira I., Sueiro R.A., Lamas J., Leiro J.M. (2017). Combined antiparasitic and anti-inflammatory effects of the natural polyphenol curcumin on turbot scuticociliatosis. J. Fish Dis..

[50] Santos D.S.D., Klauck V., Theisen C., Bordigon B., Farina R., Pereira W.A., Silva A.S.D. (2022). Addition of açai oil during the close-up dry period of Holstein cows improves colostrum quality and immune responses of their calves. An. Acad. Bras. Cienc..

[51] Santos D.S.D., Klauck V., Souza C.F., Baldissera M.D., Theisen C., Bordignon B., Silva A.S.D. (2020). Effects of the inclusion of açai oil in diet of prepartum Holstein cows on milk production, somatic cell counts and future lactation. An. Acad. Bras. Cienc..

[52] Jungbauer A., Medjakovic S. (2012). Anti-inflammatory properties of culinary herbs and spices that ameliorate the effects of metabolic syndrome. Maturitas.

[53] Van Saun R.J. (2006). Metabolic profiles for evaluation of the transition period. American Association of Bovine Practitioners Conference Proceeding.

[54] Silva P.R.L. (2009). Perfil Sanguíneo de Fêmeas Bovinas em Gestação e no Periparto e Avaliação da Transferência de Imunidade Passiva aos Descendentes. Master’s Dissertation.

[55] Tóthová C.S., Nagy O., Seidel H., Konvičná J., Farkašová Z., Kováč G.J.A.V.B. (2008). Acute phase proteins and variables of protein metabolism in dairy cows during the pre-and postpartal period. Acta Vet. Brno.

[56] Lee W.-C., Hsiao H.-C., Wu Y.-L., Lin J.-H., Lee Y.-P., Fung H.-P., Chen H.-H., Chen Y.-H., Chu R.-M. (2003). Serum C-reactive protein in dairy herds 102–107. Can. J. Vet. Res..

[57] Thomas F.C., Waterston M., Hastie P., Parkin T., Haining H., Eckersall P.D. (2015). The major acute phase proteins of bovine milk in a commercial dairy herd. BMC Vet. Res..

[58] Szczubial M., Dabrowski R., Kankofer M., Bochniarz M., Komar M. (2012). Concentration of serum amyloid A and ceruloplasmin activity in milk from cows with subclinical mastitis caused by different pathogens. Pol. J. Vet. Sci..

[59] Kaya S., Merhan O., Kacar C., Colak A., Bozukluhan K. (2016). Determination of ceruloplasmin, some other acute phase proteins, and biochemical parameters in cows with endometritis. Vet. World.

[60] Barbosa K.B.F. (2010). Oxidative stress: Concept, implications and modulating factors. Rev. Nutr..

[61] Moretti D.B., Santos C.B., Alencar S.M., Machado-Neto R. (2020). Colostrum from primiparous Holstein cows shows higher antioxidant activity than colostrum of multiparous ones. J. Dairy Res..

[62] Urh C., Denißen J., Gerster E., Kraus N., Stamer E., Heitkönig B., Spiekers H., Sauerwein H. (2019). Short communication: Pro- and antioxidative indicators in serum of dairy cows during late pregnancy and early lactation: Testing the effects of parity, different dietary energy levels, and farm. J. Dairy Sci..

[63] Corset A., Remot A., Graulet B., Poton P., Philau S., Ricouleau J., Dhumez O., Germon P., Boudon A., Boutinaud M. (2024). Effects of parity and week after calving on the metabolic, redox, and immune status of dairy cows. J. Dairy Sci..

